# How do AI micro-dramas lead to addictive use among older adults? A chain mediation model of escapism motivation and flow experience

**DOI:** 10.3389/fpsyg.2026.1815140

**Published:** 2026-06-24

**Authors:** Wei Li, Shijing Cheng, Jiwei Xiao, Heng Lan, Xiang Li

**Affiliations:** 1 Zhang Daqian School of Fine Arts, Neijiang Normal University, Neijiang, China; 2 School of Ceramic Art and Design Art, Jingdezhen University, Jingdezhen, China; 3 Department of Ceramic Art and Engineering, Jingdezhen Ceramic Technician College, Jingdezhen, China; 4 College of Culture Arts and Tourism, Neijiang Vocational and Technical College, Neijiang, China; 5 College of Arts and Media, Hubei Business College, Wuhan, China

**Keywords:** addictive use, AI micro-dramas, algorithmic personalization, emotional realism, escapism motivation, flow experience

## Abstract

Generative AI is deeply involved in the production and recommendation distribution of micro-short dramas, allowing older adult users to face a higher risk of out-of-control use while obtaining emotional compensation and immersive experience. This study mainly examines how three types of characteristics of AI micro-drama content (emotional realism, narrative involvement, and algorithmic personalization) affect the addictive use of older adults, and examines the chain mediation model between escapism motivation and flow experience. A total of 658 valid samples were obtained by questionnaire survey. SmartPLS 4 was used for data analysis to test the measurement and structural model, and the direct and indirect effects were estimated by Bootstrap. The results show that the three types of characteristics can not only directly and positively predict addictive use, but also significantly improve escapism motivation and flow experience; Escapism motivation and flow experience further positively predict addictive use, and the role of central flow is more prominent. The mediation test supports multiple single mediation and sequential mediation paths of “escapism motivation → flow experience,” revealing that older adults are more likely to experience a gradual process from “entering for escape” to “difficult to quit after immersion.” The research provides actionable governance targets for the design of age-friendly platforms and digital health interventions in the AIGC era.

## 1 Introduction

Short videos and micro-short dramas have become one of the core forms of mobile Internet content consumption. In the process of rapid evolution of this content form, AIGC is deeply involved in the planning, script generation, storyboards and visual effects of micro-short dramas, and is coupled with the platform’s recommendation and distribution mechanism to make “AI micro-dramas” (AI-led or AI-assisted micro-short dramas) show a stronger trend of large-scale and automated production. Research based on specific cases has begun to discuss how AIGC drives the creative process and traditional cultural expression strategies of micro-short dramas, providing preliminary evidence for understanding the content mechanism of “AI + micro-short dramas” (Zheng and Cui, 2025).

The older adult group is shifting from “digital margins” in the traditional sense to high-frequency users. In the United States, about 90% of adults aged 65 and older use the Internet. In China, there are more than 152 million older adult Internet users as of 2022, accounting for 14.3% of the country’s total Internet users (Center, P. R, 2024). The wider access of older adults to the Internet provides new opportunities for them to maintain social connection, obtain information and adapt emotionally, but it also exposes them to the highly sticky design of platform-based content ecology (Hunsaker et al., 2020). Studies have suggested that while short videos bring companionship and convenience to older adults, the risks of overuse and “problematic and addictive use” cannot be ignored. Empirical work targeting older adults has found that “information cocoons” and personalized recommendations in short video platforms may be associated with mental health outcomes such as depression through paths such as increasing loneliness (He et al., 2023). Research from the perspective of technical stress also pointed out that “perceived overload” in the use of short videos can significantly predict the loneliness and mental health level of older adults, and may be intertwined with the problem of short video addiction (Wen et al., 2024). Wider research on short video addiction further suggests that addictive use of short video apps is systematically associated with variables such as social isolation, attachment, and so on (Zhang et al., 2019). However, the existing evidence mostly focuses on “short video/short video App,” and there is still a lack of targeted mechanism explanation for “AI micro-dramas,” a content form that emphasizes strong plot, strong substitution, and strong personalization. Research on the older adult group often stays at the level of “usage results” or “technical risks,” and the continuous psychological process formed by their addiction (from motivation to experience to out-of-control use) still needs to be refined.

From the perspective of explanation path, existing media addiction studies often emphasize the importance of algorithmic recommendation and attention capture mechanisms, especially in algorithmic content environments represented by short video platforms (Montag et al., 2021). But for older adult users, “why are they sucked” is often not only the result of technology push, but also closely related to their specific psychological needs and emotional adjustment methods. The reason why AI micro-dramas may have stronger psychological attraction on older adults is reflected in three mutually reinforcing content characteristics: First, emotional realism, generative AI’s “anthropomorphic presentation” in expression, tone, lens language and character interaction is more likely to trigger the audience’s social attribution and emotional connection to media objects (Epley et al., 2007), thereby improving the subjective experience of “being understood and accompanied.” Second, narrative involvement. Micro-short dramas push the audience into the story world quickly with continuous reversal and high emotional beat. The narrative “transport immersion” mechanism has been proved to enhance attention focus, emotional involvement and belief in story consistency (Green and Brock, 2000); Third, algorithmic personalization, and the platform continuously optimizes recommendations based on portrait and behavioral data, so that the content is more in line with individual interests and emotional needs; Relevant studies have shown that personalized recommendations and narrowing of the information environment may have adverse effects on loneliness and depression in older adults (He et al., 2023), and are nested with the high-frequency use of short video platforms. The above features together constitute a viewing situation that is “easier to immerse in and more difficult to quit,” providing a comprehensive entrance to the content side and distribution side for understanding the addictive use of AI micro-dramas.

Based on this, this paper introduces the chain mechanism of “escapism motivation-flow experience” to explain the formation process of addictive use of AI micro-dramas by older adults. Escapism motivation theory states that when individuals face stress, loneliness, or self-imbalance, they may tend to achieve temporary disengagement from reality obsessions through immersive activities (Baumeister, 1990). In the context of Internet use, the compensatory Internet use model emphasizes that overuse often stems from the need to respond to and compensate real problems, rather than simply “the media itself is harmful” (Kardefelt-Winther, 2014). Meanwhile, flow theory regards “high concentration, pleasure, distorted sense of time, and diminished self-awareness” as the key experiences of deep immersion (Csikszentmihalyi and Csikzentmihaly, 1990). When AI micro-short dramas are more likely to trigger the psychological chain of “escape-immersion” under the combined action of emotional realism, narrative transportation and personalized recommendation, escapism motivation will prompt individuals to continue to invest in content consumption, and continuous investment is more likely to produce flow experience. In repeated reinforcement, the immediate reward brought by flow may drive the use to slide from “self-adjustment” to “out-of-control dependence,” which eventually shows the characteristics of addictive use (Miranda et al., 2023). Accordingly, this study focuses on three issues:

1. How do the key content features of AI micro-dramas (emotional realism, narrative involvement, algorithmic personalization) affect the addictive use of older adults?
2. Does escapism motivation play a mediating role between the above characteristics and addictive use?
3. Does flow experience further form a chain mediation in the path of “escapism motivation → addictive use,” thus constituting a continuous mechanism of “motivation-experience-behavior”?

In order to answer the above questions, this paper intends to construct and test a chain mediation model with escapism motivation and flow experience as the core, and uses structural equation model and Bootstrap chain mediation test to improve the robustness of the conclusion. Therefore, this paper targets older adult micro-skit users, systematically examines the impact of AI micro-dramas characteristics on addictive use, and focuses on verifying the chain mediation model of “escapism motivation-flow experience,” so as to theoretically refine the process model of older adult digital addiction, and provide a basis for platform design (such as reducing low-friction continuous playback, increasing time prompting and interruption mechanisms, and optimizing recommendation diversity) and public health interventions (such as identifying people with high escapism motivation, and providing alternative offline support) in practice (Huang et al., 2025).

The structure of this paper is as follows: The first part introduces the research background, concept definition and research problems; The second part reviews the literature and puts forward research models and hypotheses; The third part expounds the research methods and data analysis procedures; The fourth part shows the empirical results; The fifth part discusses theoretical and practical discussions; The sixth part summarizes the main conclusions, points out the research limitations and future research directions.

## 2 Literature review and theoretical assumptions

### 2.1 Addictive use of older adults and content characteristics of AI micro-dramas

Addictive use emphasizes “persistent use that is difficult to self-control” and its potential impairment to daily functioning. In the context of short videos, the research points out from the perspective of social technology and attachment that the entertainment, personalization and other elements of the platform will push up the risk of short video application addiction through psychological adhesion mechanisms such as “site/interpersonal attachment” (Zhang et al., 2019). For older adults, digital media may not only provide companionship and emotional regulation, but also be used as a compensatory channel. Empirical studies have found that the problematic social media use of older adults is significantly related to higher subjective social isolation, suggesting that they are more likely to form high viscosity or even disordered use driven by emotional and social needs (Meshi et al., 2020). Therefore, under the background that AI micro-dramas have become an important form of content supply, it is necessary to explain the formation of addictive use by older adults from the path of “content characteristics, psychological adhesion, and behavioral consequences.”

This study summarizes the key content characteristics of AI micro-dramas into three types of perceivable stimuli: emotional realism, narrative involvement and algorithmic personalization. Emotional realism refers to the subjective realism of “like a real person” brought by the expression, intonation and emotional rhythm of the character generated by AI. Media psychology has long pointed out that people will have social reactions to media objects; When the system presents more “human-like” clues, anthropomorphic processing is more likely to occur, and emotional connection and psychological closeness are enhanced (Reeves and Nass, 1996). The classical theory of “pseudo-social interaction” also suggests that the audience may form a one-way but intimate relationship with media characters, which provides the psychological basis for continuous investment and repeated consumption (Horton and Wohl, 1956). Therefore, the stronger the emotional realism, the more likely it is to enhance the tendency of older adult users to continue watching and be difficult to quit by strengthening emotional attachment and companionship.

Narrative involvement emphasizes the degree to which the audience is drawn to the story and “enters” the narrative world. Narrative transport theory states that immersion in a story takes away attention resources, enhances emotional involvement, and weakens monitoring of external cues, thus making it more difficult for individuals to interrupt ongoing media activities (Green and Brock, 2000). AI micro-dramas use high plot density and continuous hooks to promote rapid substitution, which may be easier to trigger a viewing cycle of “short-term high-intensity immersion-continuous update,” thereby increasing the risk of addictive use. Algorithmic personalization continuously optimizes content matching through user portraits and feedback loops, making viewing costs lower and rewards more immediate. Research on short video platforms points out that platform design and content distribution mechanism may be related to adverse behavioral consequences, and personalized information flow is one of the important clues to understand high user investment (Montag et al., 2021). Research on short video addiction has found that users’ high concentration on media and content is closely related to addictive behavior (Qin et al., 2022). At the same time, short video application research also regards the recommendation algorithm as one of the key affordances, and discusses its role in the framework of “affordances, experience, and problem use” (Huang et al., 2025). Based on this, it can be inferred that when the algorithm is more personalized, older adult users are more likely to continue to obtain “tasty” content and instant gratification, reducing the probability of active stopping and switching, thereby pushing up the level of addictive use.

To sum up, the emotional realism, narrative involvement and algorithmic personalization of AI micro-dramas may promote the addictive use of older adults by enhancing psychological adhesion. Therefore, the following hypotheses are proposed:

*H1a*: The emotional realism of AI micro-dramas positively affects the addictive use of older adults.

*H1b*: The narrative involvement of AI micro-dramas plays positively affects the addictive use of older adults.

*H1c*: AI micro-drama algorithmic personalization positively affects the addictive use in older adults.

### 2.2 Escapism motivation and content characteristics of AI micro-dramas

Escape is usually regarded as a coping strategy for individuals to turn to alternative activities in order to temporarily escape negative experiences in situations of stress, loneliness or self-exhaustion (Baumeister, 1990). When online entertainment is used to alleviate reality distress and occurs repeatedly in a highly accessible, highly reinforced digital environment, it is more likely to accumulate into out-of-control and problematic use (Kardefelt-Winther, 2014). Consistent with this, the analysis of problematic use of short videos also shows that motivations such as escapism and stress release are one of the important drivers to explain problematic use of short videos (Chen et al., 2024).

Emotional realism may stimulate escape needs by enhancing social presence and quasi-social companionship. People project humanoid cues onto media objects and generate anthropomorphic responses (Epley et al., 2007), and may form “intimate hallucinatory” pseudo-social relationships with characters (Horton and Wohl, 1956). When AI micro-dramas interact with more natural expressions, tones and emotional beats, viewing is more likely to be experienced as a low-cost emotional refuge, thereby increasing the intensity of motivation to “temporarily escape reality by watching” (Epley et al., 2007). The narrative involvement directly corresponds to the subjective representation of escape experience through the psychological transport mechanism of “being taken away by the story”. The transit state emphasizes the aggregation of attention, imagination and emotion, which makes individuals temporarily break away from the present situation and enter the narrative world (Green and Brock, 2000). The high-density plot and continuous update structure of vertical screen micro-short dramas can form strong cognitive occupation and emotional traction in a short period of time, making it easier for older adult audiences to withdraw from real problems, thereby enhancing the motivation to escape. Algorithmic personalization may transform the occasional need of “wanting to escape” into a more stable motivational orientation. The emotion management view holds that individuals will choose media content to regulate emotions and obtain a more affordable psychological state (Zillmann, 1988). When the platform continues to push drama clips that are more in line with the gap between interest and emotion based on portraits, the “search, trial and error” required for escape is compressed, and the reinforcement reward is more predictable, thereby increasing the probability of watching as a coping strategy. Empirical studies also show that weakening personalization will reduce the frequency and duration of short video usage and improve self-regulation, suggesting that algorithmic personalization plays a key role in maintaining high engagement (Dekker et al., 2025).

To sum up, the emotional realism, narrative involvement and algorithmic personalization of AI micro-dramas may stimulate the motivation of older adults to escape from reality by watching short plays by enhancing emotional connection, emotional regulation and cognitive immersion. Therefore, the following hypotheses are proposed:

*H2a*: The emotional realism of AI micro-dramas plays positively affects escapism motivation.

*H2b*: The narrative involvement in AI micro-dramas positively affects escapism motivation.

*H2c*: AI micro-dramas algorithmic personalization positively affects escapism motivation.

### 2.3 AI micro-dramas content characteristics and flow experience

Flow experience refers to an individual’s high state of concentration, immersion, and pleasure in activities, often accompanied by reduced self-awareness and distorted sense of time (Csikszentmihalyi and Csikzentmihaly, 1990). In digital media research, flow is regarded as the key psychological mechanism of “high-viscosity use,” and the interactivity and presence of online environments can enhance focus and immersion, thus shaping a more “addictive” experience (Novak et al., 2000). Research on short video applications further shows that platform availability can affect problematic use through flow (Huang et al., 2025), while in the TikTok scenario, users’ “psychological focus and immersion” in media and content is closely related to addiction behavior (Qin et al., 2022). It can be inferred from this that the key content features of AI micro-dramas can be understood as the “stimulus source” that triggers flow, and may significantly improve the immersion level of older adult users. Emotional realism improves anthropomorphic processing and emotional involvement by enhancing the emotional cues and interaction sense of “like a real person,” making attention more concentrated, external interference more difficult to enter, and thus making it easier to enter the flow state. Anthropomorphic theory points out that people are more inclined to experience and respond to non-human objects “as people” when social needs and clue availability are improved (Epley et al., 2007), which provides a psychological basis for AI micro-short dramas to enhance immersion with realistic expressions, intonation and emotional rhythm. Narrative involvement promotes flow through narrative transport mechanisms. Narrative transport emphasizes that individuals will have stronger attention focus, imagination, and emotional engagement when they are absorbed by the story (Green and Brock, 2000), which is highly consistent with the description of “concentration, immersion, and time distortion” described by flow. The high plot density and continuous “hook” structure of AI micro-short dramas may accelerate older adult audiences’ entry into the story world, increase the intensity of immersion and the willingness to continue watching, and make it easier to generate a flow experience. Algorithmic personalization provides “continuous invested objects” for flow by reducing selection costs and improving content matching. Short video research shows that affordances such as recommendation algorithms, low-cost interactions, and multi-modal presentation can significantly induce flow and further correlate problematic use (Huang et al., 2025). At the same time, factors such as information and system quality will enhance users’ psychological focus on media and content, which will be related to addiction (Qin et al., 2022). Therefore, when AI micro-short dramas continue to push “more tasty” plots and characters driven by algorithms, older adult users are more likely to maintain a high level of concentration and immersion, and the flow experience will be enhanced accordingly.

To sum up, the emotional realism, narrative involvement and algorithmic personalization of AI micro-dramas may significantly enhance the flow experience of older adult users by improving emotional connection, cognitive immersion and content matching. Therefore, the following hypotheses are proposed:

*H3a*: The emotional realism of AI micro-skit plays positively affects the flow experience.

*H3b*: The narrative involvement in AI micro-dramas positively affects the flow experience.

*H3c*: AI micro-dramas algorithmic personalization positively affects the flow experience.

### 2.4 Escapism motivation and addictive use in older adults

Escapism motivation usually refers to the individual’s tendency to choose immersive activities in order to temporarily get rid of negative experiences in situations of stress, loneliness or self-exhaustion (Baumeister, 1990). In digital media use research, negative life situations will stimulate the motivation to “go online to alleviate negative emotions,” and this use for the purpose of coping compensation is more likely to be reinforced and turned to problematic (addictive) use in online environments with high availability and strong feedback (Kardefelt-Winther, 2014). Social network contraction and subjective social isolation are more common for older adults. Previous studies of older adult samples have also found that problematic social media use is associated with higher subjective social isolation, suggesting that the motivation of “surfing the Internet to alleviate loneliness” may be an important psychological entrance for their out-of-control use (Meshi et al., 2020). In social media addiction mechanisms research, evasion needs to be shown to drive addiction formation through pathways such as platform-induced immersion states (Miranda et al., 2023). In motivation measurements and longitudinal tests, avoidance motivation not only predicts social network addiction, but also plays a mediating role between “frequency of use and addiction (Cuadrado et al., 2022). In other words, when media use is positioned as a coping way to manage negative emotions and escape the troubles of reality, individuals are more likely to develop a dysregulated cycle of “repeated entry, difficulty in stopping, regret using but continuing,” which explains the risk of AI micro-dramas. The addiction risk provides a direct motivational basis (Miranda et al., 2023). Placing the above mechanism into the context of older adult AI micro-short dramas, the role of evading motivation may be more prominent. AI micro-dramas are characterized by low entry costs, strong plot hooks and continuous pursuit, making them a “ready-to-use” emotional refuge. The case study of “chasing and watching dramas” for older adults pointed out that continuous viewing of older adults is often related to the compensatory motivation of “managing low mood and loneliness” (Sharma et al., 2022). At the same time, systematic review and analysis also show that continuous viewing has a significant positive correlation with psychological problems such as loneliness and depression (Alimoradi et al., 2022). Therefore, when older adults tend to use AI micro-dramas as a strategy to escape loneliness, boredom, or stress, the more likely it is to accumulate into addictive use manifestations (such as loss of control, over-engagement, and impaired function).

To sum up, escapism motivation may prompt older adults to gradually form a usage pattern that is repeatedly viewed and difficult to control by driving older adults to use AI micro-dramas as a tool for emotional regulation and escape from reality. Therefore, the following hypotheses are proposed:

*H4*: Escapism motivation positively affects addictive use in older adults.

### 2.5 Flow experience and addictive use in older adults

Flow is often regarded as the proximal psychological mechanism of “high-viscosity use.” When content continues to provide clear goals, immediate feedback, and moderate challenges, users are more likely to enter a state of immersion and tend to prolong stay and repeat use (Novak et al., 2000). This is particularly critical for older adult users. When online entertainment is used to fill the emotional gap caused by loneliness, boredom or stress, flow will further weaken the monitoring of time and stop clues, making “watch another episode, brush again” more automated and inertial, thereby pushing up the risk of out-of-control use (Csikszentmihalyi and Csikzentmihaly, 1990). Existing empirical studies on short video use show that flow experience during short video viewing is significantly correlated with addictive use: for example, in the short video platform environment, flow experience is positively correlated with addictive use, and flow strengthens immersion and engagement in content (Nong et al., 2023). In the TikTok scenario, research also points out that users’ psychological focus on media and content and flow experience have a significant effect on addictive behavior. Flow has both direct effects and can indirectly affect addiction through experience paths related to system/information quality (Qin et al., 2022). More importantly, the characteristics of short video platforms will affect the short video addiction behavior of middle-aged and older adult users through psychological processes such as flow experience and social belonging, indicating that flow is not only applicable to young samples, but is a key link in the mechanism of older adult addiction (Xu et al., 2025). In addition, flow has also been repeatedly identified as an important experiential channel between motivation and addiction in social network addiction research, reinforcing the view that “immersive experience is the proximal driver of addictive behavior” (Miranda et al., 2023).

To sum up, AI micro-dramas are characterized by strong narrative traction, continuous update tracking and personalized distribution, and are more likely to continue to trigger the concentration and immersion of older adult users during the viewing process. When flow experiences occur frequently and are continuously strengthened by instant rewards, Older adult users are more likely to experience addictive use performance such as difficulty in stopping, loss of control of time, and impaired daily functions. Therefore, the following hypotheses are proposed:

*H5*: Flow experience positively affects addictive use in older adults.

### 2.6 Escapism motivation and flow experience

For the use of media with “emotional regulation” as the core, escapism motivation is not only the reason for “why you start using it,” but also the psychological precondition for “why you are easier to get sucked in.” Escape-driven media engagement is manifested in turning to alternative activities for temporary relief under real-world stress, loneliness, or negative emotions, and this behavioral tendency is more reinforced and turned to sustained engagement in long-term, high-availability online environments (Jouhki et al., 2022). In addition, motivational orientation studies have pointed out that escapism motivation is able to predict multiple overuse behaviors (including online games, Internet use, etc.) and has a significant impact on online addiction pathways (Chen et al., 2024). Related immersive experience research also shows that in video games and other digital interactions, individuals tend to experience a high degree of absorption/fluency for the purpose of escapism, and this experience state is closely related to continuous engagement and attention occupancy (Marques et al., 2023). Specifically, in immersive games such as role-playing, participants with high escapism motivation are more likely to enter a flow state than low escapers, and therefore gain a deeper sense of immersion (Larche et al., 2021), suggesting that the need to escape not only prompts individuals to participate in medium use, but also enhances the intensity of the immersion experience during use. In addition, studies in the field of social media and problematic Internet use have pointed out that there is a correlation between escapism motivation and immersive use states (including intense attention focus and loss of self-time). This mechanism helps explain why evasive users are more likely to form persistent, uncontrollable use patterns (Miranda et al., 2023). Therefore, by migrating the above mechanism to the context of AI micro-short dramas, it can be reasonably inferred that when older adults’ escapism motivation prompts them to choose to watch AI micro-short dramas, this motivation will increase their attention occupation and emotional involvement during the viewing process, making it easier for them to experience a flow state of high concentration and immersion. The continuous plot structure, instant feedback, and rich plot of AI micro-short dramas can amplify this sense of attention focus and immersion, while viewing with the goal of escape is more likely to weaken interruption clues, making the flow experience easier to occur and more difficult to interrupt.

To sum up, in the theoretical framework of this study, escapism motivation not only drives medium participation, but should also be regarded as an important psychological prefactor to trigger flow experience. Therefore, the following hypotheses are proposed:

*H6*: Escapism motivation positively affects flow experience.

### 2.7 The mediating effect of escapism motivation and flow experience

In order to explain the mechanism of “the content characteristics of AI micro-short dramas on the addictive use of older adults,” this study regards escapism motivation and flow experience as two key internal psychological processes. The perspective of compensatory use and self-determinism emphasizes that an individual’s escapism motivation due to realistic stress, loneliness, or negative emotions can drive the choice of immediately available alternative activities and form a highly viscous use pattern. There is a robust association between this motivation and problematic Internet use (Kırcaburun and Griffiths, 2019; Yildirim Demirdöğen et al., 2024). At the same time, medium affordancy structures can facilitate flow experiences during user experience, and flow itself is the proximal psychological mechanism of high immersion and attention focus, which has been shown to be closely related to uncontrolled use behaviors (Burleigh et al., 2025; Novak et al., 2000).

First of all, regarding the mediating role of escapism motivation, content characteristics such as emotional realism and narrative involvement may enhance the audience’s psychological attachment to media content, making viewing a strategy to regulate emotions or escape from real problems. Across multiple media types (such as social media, games, or online interactive environments), escapism motivation has been empirically shown to enhance users’ preferences for online activities and is significantly associated with problematic use (Yildirim Demirdöğen et al., 2024). For example, in online games, escapism motivation can predict immersion experiences and further correlate problem behaviors, supporting the need for escape as a psychological bridge between behavior initiation and reinforcement (Marques et al., 2023). Therefore, content characteristics may indirectly push up the addictive use of older adults by promoting the motivation of “watching to achieve emotional escape or psychological compensation.”

Secondly, for the intermediary logic of flow experience, the availability conditions such as continuous feedback of AI micro-short dramas, compact plot and low exit cost are conducive to the occurrence of flow states such as psychological concentration, immersion and temporal distortion, while high-level flow experiences are associated with over-engagement or disordered behavior in digital interactive use (Burleigh et al., 2025). The “sense of disappearance of time” and “high concentration of attention” in the flow state are typical psychological characteristics of problematic use (Zheng, 2023). If older adult users enter the flow state more frequently based on content characteristics, they are more likely to show continuous viewing behavior and usage patterns that are difficult to interrupt.

Based on the above theoretical and empirical support, this study further proposes a chain mediation model: the content characteristics of AI micro-short dramas not only affect addictive use by enhancing escapism motivation, but may also enhance the subjects’ flow experience through escapism motivation, making flow an important connecting pathway from motivation to addictive behavior. Previous studies on digital games have shown that evasive users are more likely to reach the streaming state under higher immersion conditions, resulting in stronger use persistence and behavioral tendencies to lose control (Burleigh et al., 2025; Marques et al., 2023). The series mediation path of escapism motivation and flow experience is helpful to get close to the real psychological mechanism formed by the addiction of AI micro-short dramas in older adults. Therefore, the following hypotheses are proposed:

*H7a*: Escapism motivation plays a mediating role between emotional realism of AI micro-dramas and addictive use in older adults.

*H7b*: Escapism motivation plays a mediating role between narrative involvement in AI micro-dramas and addictive use in older adults.

*H7c*: Escapism motivation mediates between AI micro-drama algorithmic personalization and addictive use in older adults.

*H7d*: Flow experience plays a mediating role between emotional realism of AI micro-dramas and addictive use in older adults.

*H7e*: Flow experience plays a mediating role between narrative involvement in AI micro-dramas and addictive use in older adults.

*H7f*: Flow experience mediates between AI micro-drama algorithmic personalization and addictive use in older adults.

*H7g*: Escapism motivation and flow experience play a chain-mediating role between emotional realism of AI micro-dramas and addictive use in older adults.

*H7h*: Escapism motivation and flow experience play a chain-mediating role between narrative involvement in AI micro-dramas and addictive use in older adults.

*H7i*: Escapism motivation and flow experience play a chain-mediating role between AI micro-drama algorithmic personalization and addictive use in older adults.

### 2.8 Research model

**Figure 1:**
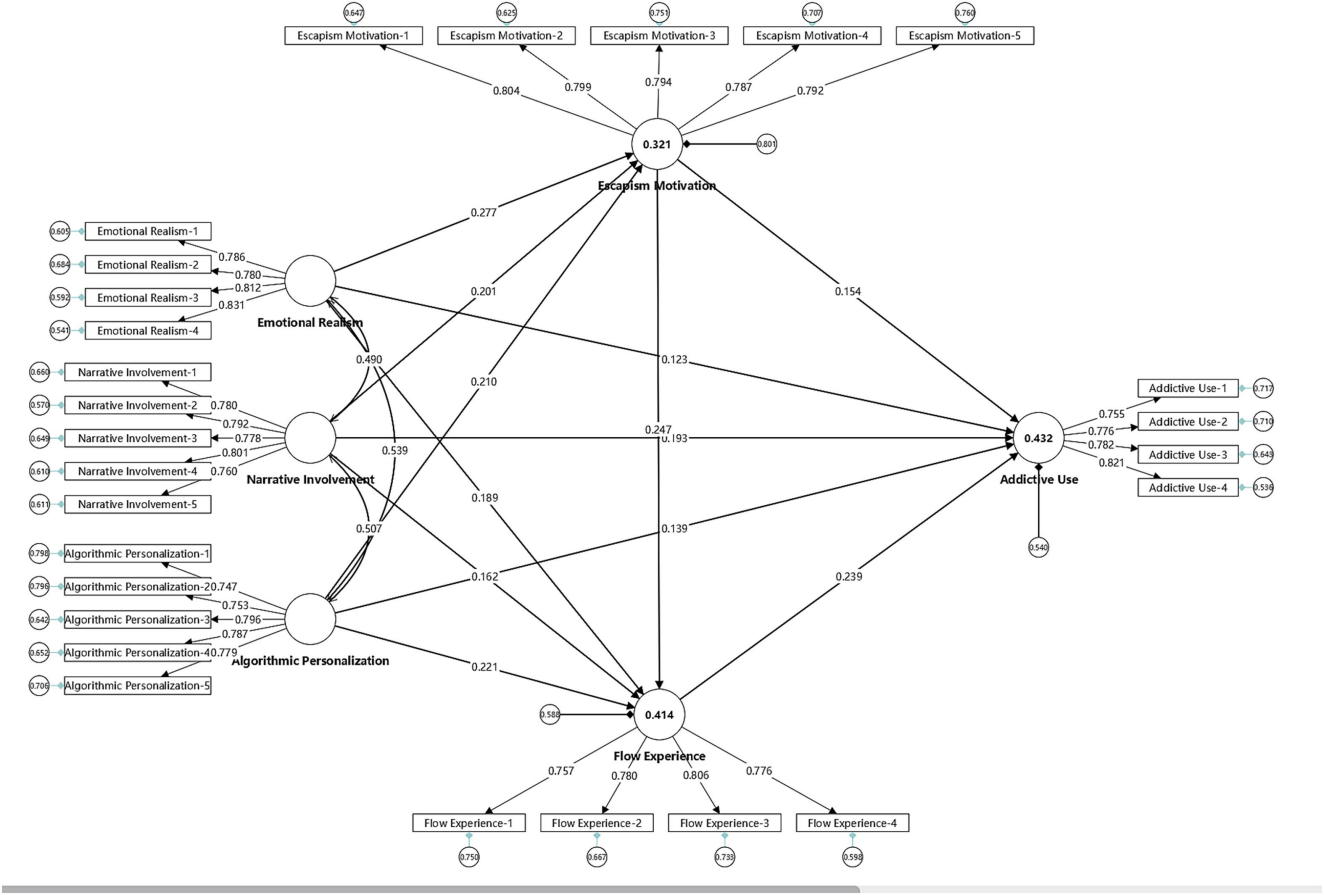
Model of the impact of AI micro-drama content characteristics on the addiction of older adults.

This study aims to explore the effect of AI micro-dramas on addictivity in older adults. Through the analysis of relevant literature, the content characteristics of AI micro-dramas are divided into three components: emotional realism, narrative involvement, and algorithmic personalization. Based on the aforementioned research hypotheses, the research model of this paper is constructed, as shown in Figure 1.

## 3 Methodology

### 3.1 Research design and research objects

This study uses a questionnaire survey design to test the chain mediation model of “content characteristics of AI micro-dramas (emotional realism, narrative involvement, algorithmic personalization) → escapism motivation → flow experience → addictive use.” The research subjects are older adults aged 60 and above, who have watched “micro-short dramas, short dramas, and short drama videos” in the past month, and self-evaluated that they have been exposed to relevant plot content produced/presented by AI (such as AI dubbing, AI special effects, AI generated screens or platform labeling “AI production, AI short drama,” etc.).

The formal survey uses the professional online platform “Questionnaire Star” (wjx.cn) to design and distribute the questionnaire, adopts the method of random sampling, and combines the offline community or senior university scene to recruit and assist in filling it out. In order to reduce the deviation caused by the difference in numerical ability, the research assistant is allowed to only explain the meaning and operation of the question (without guiding the answer) on the spot. The questionnaire is filled out anonymously and does not collect personally identifiable information. The study was approved by the ethical review of the unit before the start, and followed the principles of voluntary participation and withdrawal at any time.

### 3.2 Scale design

The six core variables involved in this study (emotional realism, narrative involvement, algorithmic personalization, escapism motivation, flow experience, addictive use) were all measured using scales. As shown in Table 1, the scale items of each variable mainly refer to the mature research in related fields, and make necessary revisions and expression optimization in combination with the specific situation and objects of this study. All scale items were measured using a 7-point Likert scale, where 1 represents “strongly disagree” and 7 represents “strongly agree,” which was used to assess the strength of the respondents’ attitudes.

**Table 1:** Questionnaire items and sources adopted.

Variables	Number	Items	Sources
Emotional realism	ER1	The characters in the AI micro-drama watched have real expressions and look like real people.	Reeves and Nass (1996), Horton and Wohl (1956)
	ER2	The emotional changes in AI micro-dramas make me feel very real.	
	ER3	I think the characters in the AI micro-drama show real emotions.	
	ER4	The intonation and emotional rhythm in the AI micro-drama brought me a strong emotional resonance.	
Narrative involvement	NI1	When I watch AI micro-dramas, I can quickly enter the world of stories.	Green and Brock (2000)
	NI2	The plot development of the AI micro-short drama makes me feel very attractive.	
	NI3	I am easily attracted to and invested in the storylines in AI micro-dramas.	
	NI4	While watching AI micro-dramas, I was able to fully focus on the story.	
	NI5	The plot of the AI micro-drama makes me feel as if I have experienced the story together with the characters.	
Algorithmic personalization	AP1	I think the content recommended by the AI micro-drama is very in line with my interests.	Montag et al. (2021), Huang et al. (2025)
	AP2	The content of AI micro-dramas is personalized and recommended based on my viewing habits.	
	AP3	I prefer to watch personalized content recommended to me by the platform.	
	AP4	I think the recommended content of AI micro-dramas is very consistent with my emotional needs.	
	AP5	Recommended AI micro-dramas usually make me find very interesting.	
Escapism motivation	EM1	I watch AI micro-dramas to temporarily escape the stress of life.	Kardefelt-Winther (2014), Chen et al. (2024)
	EM2	When I feel lonely, I will relieve my loneliness by watching AI micro-dramas.	
	EM3	Watching AI micro-dramas helped me get rid of my troubles in real life.	
	EM4	I use watching AI micro-dramas to relax myself and forget the troubles of the outside world.	
	EM5	Whenever I feel in a bad mood, I watch AI micro-dramas to regulate my emotions.	
Flow experience	FE1	When watching AI micro-dramas, I will fully devote myself to the plot and forget the things around me.	Csikszentmihalyi and Csikzentmihaly (1990), Novak et al. (2000)
	FE2	When watching AI micro-dramas, I will be highly concentrated and completely focused on the plot.	
	FE3	When watching the AI micro-drama, I was completely immersed in the characters and plot, ignoring time.	
	FE4	Watching AI micro-dramas made me feel so pleasant that I almost did not want to stop.	
Addictive use	AU1	Even if I know I should stop, I often cannot help but want to watch more AI micro-dramas.	Kardefelt-Winther (2014), Miranda et al. (2023)
	AU2	Sometimes I forget what I was supposed to do when watching AI micro-dramas.	
	AU3	Even though I knew I had been watching it for a long time, I still could not stop and continued to watch the AI micro-dramas.	
	AU4	I often feel that time flies quickly when watching AI micro-dramas.	

### 3.3 Data analysis

SPSS 26 and SmartPLS 4 software were used for data processing and analysis in this study. SmartPLS 4 was chosen mainly based on its strong support for complex structural equation modeling, rich modeling capabilities, and clear presentation of results.

The specific analysis process is as follows: First, SPSS 26 is used to make descriptive statistics on the demographic characteristics of the sample, such as gender, age, and residence, so as to fully understand the sample distribution. Secondly, the measurement model was evaluated with the help of SmartPLS 4, and the external load, Cronbach’s Alpha, combined reliability and mean variance extraction were tested to verify the reliability and validity of the scale. Finally, in the link of structural model evaluation and hypothesis testing, the Bootstrap sampling method of SmartPLS 4 is used to test the significance of path coefficient, and the explanatory power and predictive efficiency of the model are comprehensively evaluated by combining R ^2^ and Q ^2^ indicators, so as to verify the relationship between variables and the empirical support of theoretical hypotheses.

## 4 Results

### 4.1 Demographic characteristics of the respondents

Descriptive statistical analysis was conducted on 658 valid questionnaires, and the frequency analysis results showed that as shown in Table 2, 48.18% were males and 49.54% were females, and the ratio of sexes was relatively balanced (MobTech. 2020). In terms of age distribution, the 60-65-year-old group accounted for 30.85%% and the 65-70-year-old group accounted for 31.46%. The proportion of respondents in these two age groups was higher, reflecting that the sample mainly consists of older adults (Wu and Wen, 2023). In terms of living conditions, living with a spouse accounted for 50.46%, followed by living alone accounted for 25.53%, and living with children accounted for 21.73%. On the whole, the sample has certain heterogeneity in gender, age and residential form, which provides the necessary variation basis for subsequent analysis.

**Table 2:** Respondent demographic characteristics (sample size *n* = 658).

Name	Options	Frequency	Percent (%)	Cumulative percentage (%)
Gender	Other/inconvenient to disclose	15	2.280	2.280
	Female	326	49.544	51.824
	Male	317	48.176	100.000
Age	Aged 60–65	203	30.851	30.851
	Aged 65–70	207	31.459	62.310
	70–75 years old	144	21.884	84.195
	Aged 75 and above	104	15.805	100.000
Living conditions	Live with children	143	21.733	21.733
	Living with a spouse	332	50.456	72.188
	other	15	2.280	74.468
	live alone	168	25.532	100.000
View frequency in the past 1 month	Occasionally (less than 1 per week)	100	15.198	15.198
	Almost every day	165	25.076	40.274
	Sometimes (1–3 times per week)	181	27.508	67.781
	Frequent (4–6 times per week)	212	32.219	100.000
Average daily viewing time	1 h to 2 h	232	35.258	35.258
	Over 2 h	104	15.805	51.064
	30 min to 1 h	204	31.003	82.067
	Less than 30 min	118	17.933	100.000
Main viewing platform (single choice)	Bilibili	6	0.912	0.912
	other	10	1.520	2.432
	Red Note	25	3.799	6.231
	Kwai	144	21.884	28.116
	Tiktok	317	48.176	76.292
	Video number	156	23.708	100.000
Autoplay usage habits	Never	119	18.085	18.085
	occasionally	210	31.915	50.000
	Always	123	18.693	68.693
	often	206	31.307	100.000
Top reasons to watch	other	27	4.103	4.103
	Entertainment	265	40.274	44.377
	Learn/understand something	98	14.894	59.271
	Relieve loneliness/stress	268	40.729	100.000
Total		658	100.0	100.0

In terms of viewing behavior in the past month, the respondents’ contact frequency with AI micro-dramas/short drama videos was mainly medium to high frequency: “frequent” viewers 4–6 times a week accounted for the highest proportion, 32.22%, followed by “sometimes” 1–3 times a week accounting for 27.51% and “almost every day” accounting for 25.08%. Corresponding to the frequency, the average daily viewing time is mainly between 30 min and 2 h, of which “1 h to 2 h” accounts for the highest proportion at 35.26%, followed by “30 min to 1 h” accounts for 31.00%; “Less than 30 min” accounted for 17.93%; “More than 2 h” accounted for 15.81%. Platform usage (single choice) results show that Douyin is the most important viewing platform, accounting for 48.18%, followed by video accounts accounting for 23.71%, and Kuaishou accounting for 21.88%. In terms of automatic playback usage habits, “occasionally” accounts for 31.92%, and “frequently” accounts for 31.31%. The proportions of the two options are similar. In terms of viewing motivation, “relieving loneliness and stress” accounted for 40.73%, and “entertainment” accounted for 40.27%. The two options were almost listed as the main reasons. Overall, the samples show clear structural distribution characteristics in viewing intensity, platform selection, automatic playback habits and motivation, which are well in line with the chain of “continuous use-immersion experience-addiction tendency” that the research focuses on.

### 4.2 Reliability and validity analysis

According to the scales used in this survey, the reliability analysis of each item was carried out, and the results showed that the reliability of all scales was high. Specifically (Table 3), the factor load of each item is between 0.714 and 0.810, which is significantly higher than the commonly used standard (0.5), and exceeding the ideal threshold of 0.7 (Cheung et al., 2024) indicates that each item has strong explanatory power for its latent variables, which strongly supports the construct validity of the scale. Cronbach’s *α* values ranged from 0.861 to 0.895, all exceeding the conventional reliability standard (0.7) (Nunnally and Bernstein, 1994), indicating good internal consistency of the scale. The composite reliability (CR) ranged from 0.862 to 0.896, both greater than the reference value of 0.6 (Bagozzi and Yi, 1988), and the AVE value ranged from 0.597 to 0.644, both greater than the reference value of 0.5 (Hair et al., 2011), indicating that the scale has high explanatory power and good aggregate validity on each latent variable. Therefore, the scale used in this survey performed well in terms of reliability and validity, which laid a solid foundation for subsequent analysis.

**Table 3:** Reliability and validity analysis.

Construct	Item	Factor loadings	Cronbach’s alpha	CR	AVE
Emotional realism	ER1	0.810	0.879	0.879	0.644
	ER2	0.804			
	ER3	0.753			
	ER4	0.758			
Narrative involvement	NI1	0.793	0.887	0.887	0.612
	NI2	0.761			
	NI3	0.773			
	NI4	0.775			
	NI5	0.767			
Algorithmic personalization	AP1	0.731	0.880	0.881	0.597
	AP2	0.729			
	AP3	0.793			
	AP4	0.775			
	AP5	0.766			
Escapism motivation	EM1	0.786	0.895	0.896	0.632
	EM2	0.790			
	EM3	0.793			
	EM4	0.779			
	EM5	0.778			
Flow experience	FE1	0.714	0.861	0.862	0.608
	FE2	0.785			
	FE3	0.808			
	FE4	0.746			
Addictive use	AU1	0.727	0.863	0.864	0.614
	AU2	0.794			
	AU3	0.766			
	AU4	0.789			

### 4.3 Discriminant validity analysis

In this study, two complementary methods were used to test the discriminative validity of the models. One is the Fornell-Larcker criterion: it is required that the correlation coefficient between each latent variable should be less than the square root of the latent variable AVE to show that there is sufficient distinction between constructs. The second is HTMT (heterotrait-monotrait ratio): the HTMT value is lower than 0.85 as the judgment threshold (Henseler et al., 2015). Both test results meet the corresponding criteria, indicating that the measurement model of this study has good discriminatory validity. The relevant statistical results are shown in Tables 4, 5.

**Table 4:** Distinctive validity analysis (Fornell–Larcker).

Item	1	2	3	4	5	6
Addictive use	0.784					
Algorithmic personalization	0.498	0.772				
Emotional realism	0.489	0.539	0.803			
Escapism motivation	0.486	0.461	0.488	0.795		
Flow experience	0.544	0.519	0.508	0.513	0.780	
Narrative INVOLVEMENT	0.506	0.507	0.490	0.443	0.476	0.782

**Table 5:** Discrimination validity analysis (HTML).

Item	1	2	3	4	5	6
Addictive use						
Algorithmic personalization	0.499					
Emotional realism	0.490	0.540				
Escapism motivation	0.487	0.463	0.484			
Flow experience	0.551	0.524	0.506	0.518		
Narrative involvement	0.507	0.508	0.486	0.443	0.476	

### 4.4 Multicollinearity analysis

Before entering the structural model test, this study first diagnosed the risk of multicollinearity among latent variables to ensure the stability and interpretability of regression path estimation. The results of the collinearity test showed that the variance inflation factor (VIF) of each construct ranged from 1.481 to 1.586, which was less than 5 (Hair et al., 2011). The tolerance ranges from 0.630 to 0.675. Overall, the VIF is at a low level and the tolerance is higher than the common warning threshold, indicating that there is no serious linear dependence between the respective variables. In other words, variables such as emotional realism, narrative involvement, algorithmic personalization, escapism motivation and flow experience can provide relatively independent information contributions in the model, providing a reliable statistical premise for subsequent path coefficient estimation and mediation effect test (Table 6).

**Table 6:** Collinearity diagnosis.

Item	VIF	Tolerance
Emotional realism	1.485	0.673
Narrative involvement	1.499	0.667
Algorithmic personalization	1.545	0.647
Escapism motivation	1.481	0.675
Flow experience	1.586	0.630
Addictive use	1.561	0.640

### 4.5 Path analysis

**Figure 2:**
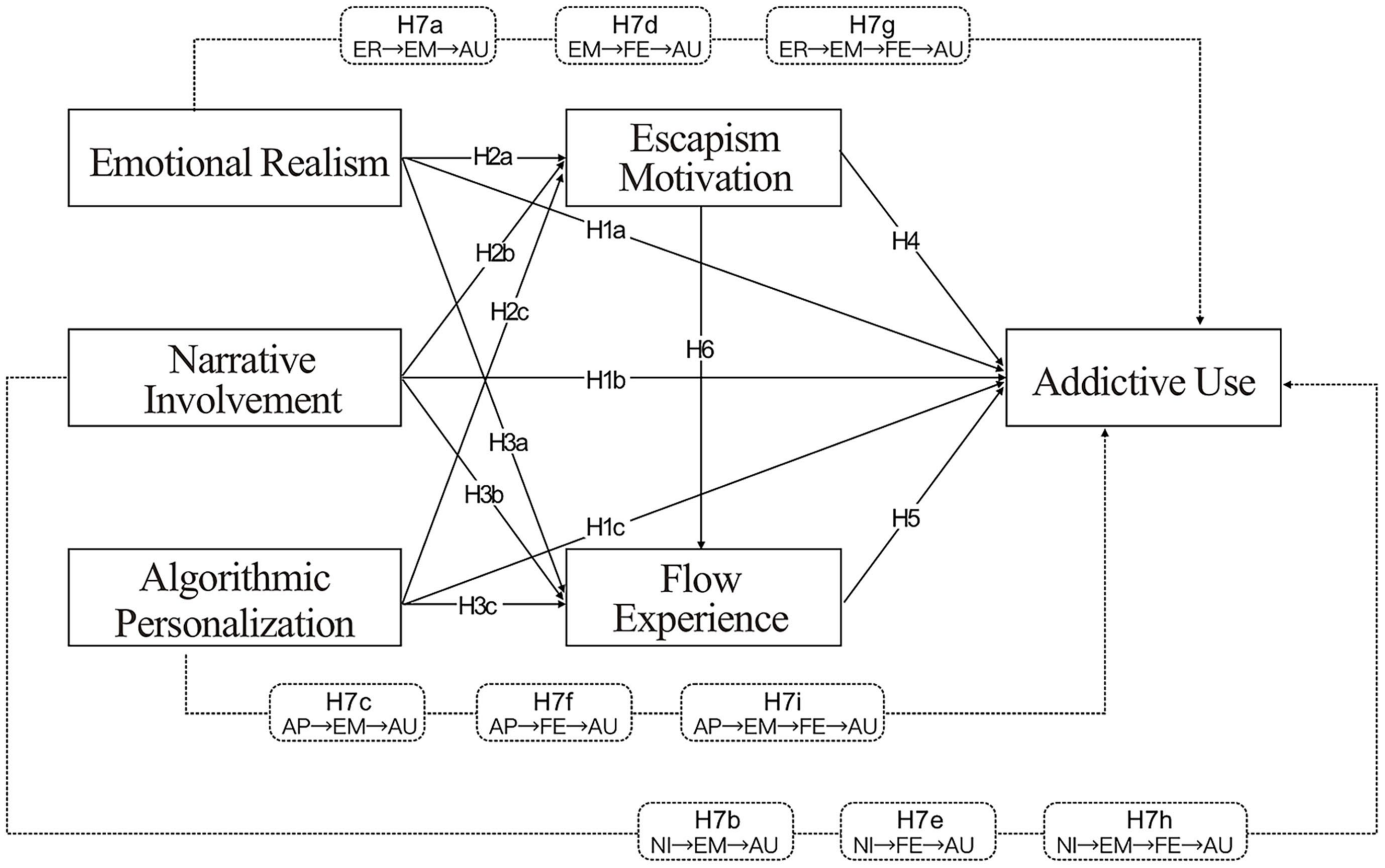
Model analysis results. ER, emotional realism; NI, narrative involvement; AP, algorithmic personalization; EM, escapism motivation; FE, flow experience; AU, addictive use.

The path coefficient of the structural model is evaluated by SmartPLS4. When the t value of the path coefficient is greater than 1.96 (Bagozzi and Yi, 1988), it means that the path coefficient passes the test at the significance level of 5% and is significant. The analysis results are shown in Table 7 and Figure 2. The details are as follows:

ER (*β* = 0.123, T = 2.326, *p* = 0.020), NI (*β* = 0.193, T = 3.818, *p* = 0.000), and AP (*β* = 0.139, T = 2.662, *p* = 0.008) versus AU Has a significant positive effect, so the assumptions H1a, H1b and H1c hold true.
ER (*β* = 0.277, T = 5.992, *p* = 0.000), NI (*β* = 0.201, T = 4.170, *p* = 0.000), and AP (*β* = 0.210, T = 4.214, *p* = 0.000) versus AU Has a significant positive effect, so the assumptions H2a, H2b, and H2c hold.
ER (*β* = 0.189, T = 3.657, *p* = 0.000), NI (*β* = 0.162, T = 3.478, *p* = 0.001), and AP (*β* = 0.221, T = 4.551, *p* = 0.000) versus AU Has a significant positive effect, so the assumptions H3a, H3b, and H3c hold.
EM (*β* = 0.154, T = 2.981, *p* = 0.003) has a significant positive effect on AU, so hypothesis H4 holds.
FE (*β* = 0.239, T = 4.529, *p* = 0.000) has a significant positive effect on AU, so the hypothesis H5 holds.
EM (*β* = 0.247, T = 5.272, *p* = 0.000) has a significant positive effect on SC, so hypothesis H6 holds.
Furthermore, EM showed a significant difference in ER versus AU (*β* = 0.043, T = 2.538, *p* = 0.014), NI versus AU (*β* = 0.031, T = 2.523, *p* = 0.009), AP versus AU (*β* = 0.032, T = 2.348, *p* = 0.010). The assumptions of H7a, H7b and H7c are true.
FE was found in ER versus AU (*β* = 0.045, T = 2.664, *p* = 0.017), NI versus AU (*β* = 0.039, T = 2.719, *p* = 0.014), AP versus AU (*β* = 0.053, T = 3.167, *p* = 0.027) Play a mediating role between them, assuming that H7d, H7e and H7f hold true.
EM and ER were found in NA versus AU (*β* = 0.016, T = 2.921, *p* = 0.007), ER versus AU (*β* = 0.012, T = 2.764, *p* = 0.004), ER versus AU (*β* = 0.012, T = 2.681, *p* = 0.005) There is a sequence mediation effect between them, and the assumption that H7g, H7h and H7i hold.

**Table 7:** Path analysis.

Path	*β*	STDEV	T	*p*	Decision		
					2.5%	97.5%	Decision
(H1a): Emotional realism → Addictive use	0.123	0.053	2.326	0.020	0.014	0.231	Supported
(H1b): Narrative involvement → Addictive use	0.193	0.051	3.818	0.000	0.084	0.280	Supported
(H1c): Algorithmic personalization → Addictive use	0.139	0.052	2.662	0.008	0.035	0.235	Supported
(H2a): Emotional realism → Escapism motivation	0.277	0.046	5.992	0.000	0.196	0.376	Supported
(H2b): Narrative involvement → Escapism motivation	0.201	0.048	4.170	0.000	0.102	0.289	Supported
(H2c): Algorithmic personalization → Escapism motivation	0.210	0.050	4.214	0.000	0.099	0.301	Supported
(H3a): Emotional realism → Flow experience	0.189	0.052	3.657	0.000	0.073	0.279	Supported
(H3b): Narrative involvement → Flow experience	0.162	0.047	3.478	0.001	0.082	0.262	Supported
(H3c): Algorithmic personalization → Flow experience	0.221	0.049	4.551	0.000	0.124	0.310	Supported
(H4): Escapism motivation → Addictive use	0.154	0.052	2.981	0.003	0.061	0.252	Supported
(H5): Flow experience → Addictive use	0.239	0.053	4.529	0.000	0.134	0.329	Supported
(H6): Escapism motivation → Flow experience	0.247	0.047	5.272	0.000	0.160	0.346	Supported
(H7a): Emotional realism → Escapism motivation → Addictive use	0.043	0.017	2.538	0.011	0.014	0.079	Supported
(H7b): Narrative involvement → Escapism motivation → Addictive use	0.031	0.012	2.523	0.012	0.009	0.055	Supported
(H7c): Algorithmic personalization → Escapism motivation → Addictive use	0.032	0.014	2.348	0.019	0.010	0.060	Supported
(H7d): Emotional realism → Flow experience → Addictive use	0.045	0.017	2.664	0.008	0.017	0.082	Supported
(H7e): Narrative involvement → Flow experience → Addictive use	0.039	0.014	2.719	0.007	0.014	0.068	Supported
(H7f): Algorithmic personalization → Flow experience → Addictive use	0.053	0.017	3.167	0.002	0.027	0.089	Supported
(H7g): Emotional realism → Escapism motivation → Flow experience → Addictive use	0.016	0.006	2.921	0.004	0.007	0.028	Supported
(H7h): Narrative involvement → Escapism motivation → Flow experience → Addictive use	0.012	0.004	2.764	0.006	0.004	0.021	Supported
(H7i): Algorithmic personalization → Escapism motivation → Flow experience → Addictive use	0.012	0.005	2.681	0.008	0.005	0.022	Supported

### 4.6 Model explanatory power and predictive ability

From the perspective of explanatory power and predictive power of the model (shown in Table 8), the determination coefficient (R^2^) of the core endogenous variables is at a moderately high level. When the R ^2^ value is greater than 0.25 (Sarstedt et al., 2014), it means that the model has a stronger explanatory ability to endogenous variables. The R ^2^ of addictive use is 0.432, the R^2^ of flow experience is 0.414, and the R^2^ of escapism motivation is 0.321, indicating that the model can explain about 43.2% of the variance of addictive use, and also has strong explanatory power for the variation between flow experience and escapism motivation. Further prediction test results show that the Q^2^ predict of the three endogenous variables are all positive (0.261–0.297). If Q^2^ > 0, it means that the model has predictive correlation with endogenous variables (Hair et al., 2011). The corresponding error indicators (RMSE = 0.841–0.862; MAE = 0.672–0.697) are also in the acceptable range. Generally speaking, the structural model not only performs well at the fitting level, but also has solid statistical support at the interpretation and prediction levels, which can provide sufficient empirical basis for the research conclusion.

**Table 8:** Model interpretation and predictive power.

Item	Q^2^ predict	RMSE	MAE	R^2^
Addictive use	0.292	0.844	0.672	0.432
Escapism motivation	0.261	0.862	0.697	0.321
Flow experience	0.297	0.841	0.676	0.414

## 5 Discussion and implications

### 5.1 Discussion

Focusing on “how the content/system characteristics of AI micro-dramas promote the addictive use of older adults,” this study constructs and tests a chain mediation model centered on escapism motivation and flow experience. The structural path results show that the three key antecedent variables (emotional realism, narrative involvement and algorithmic personalization) can significantly improve the two core psychological mechanism variables (escapism motivation and flow experience) at the same time, and have different degrees of direct impact on addictive use. At the same time, both escapism motivation and flow experience significantly positively predicted addictive use, and the central flow experience had the most prominent effect on addictive use (*β* = 0.239). This shows that older adults’ addiction to AI micro-dramas is not simply triggered by “good-looking content,” but more like a psychological to behavioral process driven by system reinforcement and emotional compensation: antecedent characteristics improve the individual’s escapism orientation and immersion experience, and then Push up continuous viewing and out-of-control use.

From the perspective of direct effects, all three kinds of antecedents have significant positive predictions on addictive use, but there are differences in intensity and mode of action. The direct effect of narrative involvement on addictive use is high (*β* = 0.193), indicating that high plot density and continuous hook structure can lock attention and promote catching up in a short time; At the same time, its significant effect on escapism motivation and flow experience (*β* = 0.201; *β* = 0.162) means that narrative not only “attracts people,” but also transforms the viewing experience into a psychological channel that is temporarily divorced from reality. Algorithmic personalization not only directly affects addictive use (*β* = 0.139), but also significantly improves escapism motivation and flow experience (*β* = 0.210; *β* = 0.221), and forms a chain indirect effect (*β* = 0.012). This suggests that the recommendation mechanism not only “improves convenience” in older adult addiction, but also is more likely to solidify the occasional viewing demand into stable high-frequency use by reducing the selection cost and enhancing the predictability of rewards. Emotional realism also shows a stable and systematic effect: it has one of the strongest effects on escapism motivation (*β* = 0.277), and simultaneously improves flow experience (*β* = 0.189) and addictive use (*β* = 0.123), and has the largest chain mediation effect (*β* = 0.016). This shows that emotional realism is more likely to transform viewing into an experience of “companionship/understanding/solace,” thus activating the escape orientation more directly in the context of emotional gaps in older adults, and further promoting immersion and loss of control.

From the perspective of a single mediation path, both algorithmic personalization and emotional realism have significant indirect effects of “flow experience → addictive use” (*β* = 0.053; *β* = 0.045), indicating that they can also directly increase the risk of addiction by enhancing the immersion experience without escapism motivation. At the same time, there is a significant path of “escapism motivation → addictive use” in emotional realism (*β* = 0.043), which means that its influence on addictive use has more obvious emotional compensation attributes. On the whole, the risk of AI micro-dramas to older adults does not come from a single factor, but from the coupling of “emotional simulation-narrative hook-algorithm enhancement”: emotional clues provide psychological comfort, narrative structure provides continuous input, and algorithm mechanism Provide continuous reinforcement, thereby jointly pushing “wanting to see” to “unable to stop.”

Further mediation checks not only verify multiple single mediation paths, but also support critical chain mediation paths. First of all, “narrative involvement → flow experience → addictive use” is significant (*β* = 0.039), indicating that narrative involvement promotes the formation of “unstoppable” use patterns by promoting concentration and immersion. Secondly, “escapism motivation → flow experience → addictive use” was significant (*β* = 0.059), showing that escapism motivation not only directly drives addictive use (*β* = 0.154), but also further amplifies risk by enhancing flow. More importantly, all three chain mechanisms are established: narrative involvement, algorithmic personalization, and emotional realism have significant indirect effects on addictive use through the sequence path of “escapism motivation → flow experience” (chain *β* = 0.012/0.012/0.016). Together, this set of evidence points to a more complete and realistic evolutionary logic: under the stimulation of content, older adults first produce the compensatory orientation (escape) of “watching to relieve loneliness, boredom or stress,” and then enter the immersion state (flow) of “time disappearing and attention converging” during viewing, and finally forms a runaway cycle (addictive use) that is more difficult to interrupt. Therefore, compared with a single intermediary, the sequence mechanism has “extra explanatory power,” which can explain why older adults not only “watch for a long time,” but also “repeatedly enter immersion and gradually lose control.”

### 5.2 Revelation

From the perspective of the process of “content characteristics-motivation and experience processing-behavioral results,” this study clarifies that the addictive use of older adults is not only driven by content attraction or algorithm reinforcement, but through the continuous psychological chain of “escapism motivation → flow experience” is gradually amplified and solidified. The structural path and intermediary test together show that algorithmic personalization, emotional realism and narrative involvement not only directly enhance addictive use, but also activate the compensatory needs (escape) of older adults first, and then push them into a stronger immersion state (flow), eventually forming an out-of-control cycle. Especially the significance of chain mediation: compared with the single mediation model, the sequence mechanism can provide additional explanatory power and better reveal the evolutionary process of “entering to relieve emotions-difficult to exit due to immersion,” thus providing a more dynamic theoretical framework for understanding media addiction in older adults.

The research results suggest that risk management for older adults should focus on the two key links of “system strengthening” and “emotional compensation” at the same time: on the one hand, the platform needs to strengthen stop clues and interruption mechanisms (such as viewing duration reminders, automatic continuous broadcast default shutdown, and continuous viewing cooling prompts) to offset the distortion of time sense and stop difficulties caused by flow; At the same time, a risk-sensitive strategy is introduced at the recommendation system level. When high-risk patterns such as long-term viewing at night and repeated viewing of similar emotional content are identified, the strengthening intensity is reduced and the content diversity is improved to avoid the algorithm’s continuous amplification of immersion and escape. On the other hand, families and communities should alleviate the motivation to escape from the source, and reduce the dependence of older adults on micro-short dramas as the main emotional compensation channel by increasing offline social interaction, interest activities and emotional support; And cooperate with concise digital literacy and self-regulation training to help them establish viewing plans and alternative activities, thereby reducing the probability of addictive use.

## 6 Conclusion, limitations, and future research

### 6.1 Conclusion

This study focuses on three types of content characteristics of AI micro-dramas (emotional realism, narrative involvement, and algorithmic personalization) to explore how they affect the addictive use of older adults through escapism motivation and flow experience. Structural path test shows that the three types of antecedents can not only directly and significantly predict addictive use, but also significantly improve escapism motivation and flow experience, indicating that the impact of AI micro-dramas is not a single “attraction” effect, but a compound mechanism of “emotional compensation-immersion reinforcement.” Furthermore, both escapism motivation and flow experience have significant positive predictive effects on addictive use, and the central flow experience has the most prominent effect on addictive use, and escapism motivation not only directly affects addictive use, but also significantly promotes flow experience, revealing the progressive relationship between motivation and experience. The mediation effect test shows that multiple single mediation and chain mediation paths are significant and the confidence interval does not contain 0. In particular, the sequence mechanism of “antecedent characteristics → escapism motivation → flow experience → addictive use” is supported, indicating that older adults Addicted to AI micro-dramas are more likely to experience a gradual process from “entering for escape” to “difficult to exit after immersion.” Overall, this study constructs and verifies a relatively clear transmission chain, provides process evidence for understanding the addiction risk of older adults in the AIGC content environment, and also provides actionable intervention targets for platform governance and active aging practices.

### 6.2 Limitations

Despite consistent and robust statistical support for this study, the following limitations exist. First, this study uses cross-sectional questionnaire data. Although the results of chain mediation are theoretically reasonable, it is still difficult to make strict causal inference. The relationship between variables may have reverse paths or dynamic mutual feedback. Secondly, the core variables mainly come from self-reporting measurement, which may be influenced by social approval, recall bias and common method bias; although it can be mitigated by procedural control and statistical testing, method effects cannot be completely ruled out. Third, the definition of “AI micro-short drama” in this study is based on participants’ self-evaluation and platform use experience, and fails to subdivide the specific types and intensity of AI generation/AI assistance (such as AI dubbing, AI roles, AI storyboards, AI interactive plots, etc.), so a more detailed explanation of the differentiated impact of different AI elements cannot be given. Fourth, the sample may be affected by regional, digital literacy and platform ecological differences, resulting in boundaries in external validity; In addition, differences within the older adult group (such as 60–69 vs. 70 +, living alone vs. non-living alone, low vs. high digital literacy) may trigger heterogeneous mechanisms, and this study has not fully developed group tests.

### 6.3 Future research directions

Based on the above limitations, future research can be deepened from the following directions. First, it is recommended to use longitudinal tracking, empirical sampling (EMA) or experimental research to enhance causal recognition, such as manipulating algorithmic personalization strength, emotional realistic cues or narrative hook density, and to observe the dynamic evolution of short-term immersion and long-term out-of-control. Second, encourage the introduction of multi-source data and objective behavior indicators, such as platform viewing time logs, night usage records, continuous updates, etc., and cross-validate with self-reported data to improve measurement accuracy and reduce common method deviations. Third, in the future, the generative elements and interactive forms of AI micro-dramas can be further subdivided, and an “AI feature pedigree” (such as anthropomorphic speech, emotional feedback, interactive plot, generative characters, etc.) can be constructed to test the differentiated path of different AI elements through escapism motivation and flow experience, thereby providing a basis for more accurate content governance. Fourthly, it is recommended to carry out research on heterogeneity and situational boundaries: for example, comparing the strength of mechanisms among older adults of different ages, different levels of loneliness and social support, and different groups with digital literacy and self-control ability; We can also further examine the buffering effect (moderation) of external factors such as family companionship, community participation, and platform usage norms to form an integrated model of “risk mechanism-protection factors.” Fifth, from the application level, research conclusions can be translated into testable intervention programs, such as randomized controlled evaluations of “stop cue design,” “cooling mechanism,” “risk-sensitive recommendation” and “digital literacy training” to test whether these strategies can effectively reduce addictive use and promote healthier digital participation in older adults.

## Data Availability

The original contributions presented in the study are included in the article/Supplementary material, further inquiries can be directed to the corresponding author.
